# Predictors of Dietitian Referrals in Hospitals

**DOI:** 10.3390/nu12092863

**Published:** 2020-09-18

**Authors:** Doris Eglseer, Silvia Bauer

**Affiliations:** Institute of Nursing Science, Medical University Graz, 8010 Graz, Austria; silvia.bauer@medunigraz.at

**Keywords:** dietitian, referral, hospital, malnutrition, nutritional counselling, nutrition therapy, nutritional support

## Abstract

Dietitian involvement has considerable benefits for hospitalized patients, resulting in better health outcomes and improved quality of life. However, dietitian referral routines are often inappropriate in hospitals. The aim of this study was to identify predictors for dietitian referrals in hospitalized patients. This study was performed on data collected in an annually conducted cross-sectional study (in the years 2017, 2018, 2019). A standardized questionnaire was used to collect data, and logistic regression and a generalized estimating equation (GEE) model were used to calculate the associations between the patient characteristics and dietitian referrals. In the final GEE model, the following predictors for dietitian referrals remained significant: diabetes diagnosis (OR 1.80), cancer diagnosis (OR 1.76), digestive disease diagnosis (OR 2.03), presence of a pressure injury (OR 1.58), risk of malnutrition based on body mass index (BMI) and weight loss (OR 1.72), risk of malnutrition based on the malnutrition universal screening tool (MUST) (2.55), and the application of any malnutrition screening at admission to hospital (2.20). Total dietitian referral rate was 16.8%. The highest rate of dietitian referrals was found in patients with a risk of malnutrition (37%). This study included a large sample of hospitalized adult patients and revealed a low dietitian referral rate among these patients. These results indicate that dietitian involvement in hospitalized patients with nutrition-related conditions urgently needs to be improved.

## 1. Introduction

Patients admitted to hospital often suffer from nutritional problems such as gastrointestinal issues, decreased appetite, or nausea. These problems can even result in malnutrition, which is a prevalent condition in hospitals, affecting up to 45% of all patients [1,2]. In older patients (65+), the prevalence of malnutrition is even higher. A recent systematic review revealed that 53% of older hospitalized patients are at risk of malnutrition, and 28% of these suffer a high risk of developing malnutrition [3]. Malnutrition primarily arises in hospital patients who suffer from acute or chronic diseases either with or without inflammatory activity, such as cancer, gastrointestinal diseases, infections, respiratory diseases, and chronic kidney disease [1,4,5]. Not only malnutrition but also overweightness/obesity are prevalent conditions in hospitalized patients [6,7].

In all patients with nutritional problems, regardless of whether they are malnourished or overweight, it is recommended that nutritional therapy and counselling be offered [8]. The nutritional care offered in hospitals includes nutritional screening, comprehensive nutritional assessment, carrying out diagnostic procedures, setting individualised goals, developing a nutritional care plan, implementing interventions, and monitoring/evaluating the effects of nutritional care [8,9]. Nutritional counselling is strongly recommended for all patients at risk of nutritional problems or nutrition-related diseases [8,9]. This counselling is provided by dietitians, who in Europe are healthcare professionals that provide nutritional care, are recognised by the national authority, and have at least an undergraduate degree. They use evidence-based approaches to support adequate nutritional practices in healthy and ill patients [10].

For individuals to receive adequate nutritional care in the hospital setting, it is critical that healthcare staff refer at-risk patients to dietitians without delay. In most countries, referrals to dietitians are conducted solely by physicians. This is based on the assumption that physicians are sufficiently trained and able to recognise a need for nutritional intervention in their patients. Unfortunately, this is often not the case in clinical practice, and a dietitian’s expertise and possible contribution to positive outcomes and wellbeing in patients is frequently underestimated by physicians [11,12]. Furthermore, if dietitians are involved in the care of patients, such referrals are made only at a very late timepoint in the hospital stay [13,14]. This has potentially negative impacts on the effectiveness of nutritional interventions and, in turn, may lead to inappropriate dietitian referral routines in hospitals.

Multidisciplinary cooperation between healthcare professionals such as dietitians, physicians, nurses, or therapists is an indispensable prerequisite to ensure adequate nutritional care in hospitals [8,15]. Dietitian referral routines are based on the referral systems used in different countries and in different institutions. It is therefore difficult to generalize referral practices. However, it is recommended that protocols are available in order to, for example, regulate how patients at risk of malnutrition are treated and when a dietitian should be involved in a patient’s therapy and care. In the UK for instance, there are specific dietetic referral criteria for adults available [16]. All registered health professionals may refer a patient to a dietitian in the UK [16]. This is not the case in other countries where only physicians are allowed to refer patients to dietitians, e.g., in Austria or the Netherlands, and defined dietetic referral criteria are a rare occurrence.

Studies have shown that involving dietitians in patient care provides these patients with considerable advantages. A systematic review about nutritional education offered in Europe to prevent diabetes, for example, revealed that dietitian-delivered interventions led to significantly greater weight reductions than interventions delivered by other healthcare personnel [17]. In patients admitted to hospital who are at risk of malnutrition, a large multicentre RCT showed that individualized nutritional support delivered by dietitians at an early timepoint offered these patients clear advantages. This support significantly lowered the risk of experiencing adverse clinical outcomes after 30 days, reduced all-cause mortality, and improved the patients’ functional status and quality of life [18]. Another systematic review found that nutritional interventions, such as prescribing oral nutritional supplements (ONS), were most effective if they were combined with dietary counselling by a dietitian [19]. Another study showed that tube-fed patients received 10% more energy and about 8% more protein from enteral formula prescribed by dietitians than formula prescribed by physicians. These findings indicate that patients are more likely to achieve their energy and protein goals if the enteral formula is prescribed by dietitians, resulting in significantly shorter hospital stays, improved albumin, and weight gain [20].

The high prevalence of nutritional problems in hospitalized patients, as well as the considerations described above, result in negative clinical outcomes such as impaired wound healing or higher complication rates, higher mortality, or diminished quality of life [21,22,23]. Involving dietitians in the care of these patients demonstrably improves their outcomes, which underlines the importance of early dietitian referrals. The systematic review of nutritional education, however, also revealed a significant knowledge gap. The demographic characteristics and medical conditions that facilitate dietitian referrals in hospitalized patients are poorly known [12]. Therefore, this study was primarily carried out to identify predictors for dietitian referrals in hospitalized patients. As a secondary aim, an assessment was performed of the referral rates to dietitians for different medical conditions in Austrian hospital settings.

## 2. Materials and Methods

### 2.1. Design

This study was performed on data collected in an annually conducted cross-sectional study to measure the quality of care offered in hospitals, including nutritional care (International Measurement of Care Quality, LPZ study). This measurement takes place annually on one specific day in November. For the analysis in this study, data were included from patients admitted to Austrian hospitals on the day of measurement in 2017, 2018, and 2019.

### 2.2. Instrument

The LPZ study uses a standardized questionnaire to collect data on demographic patient characteristics, such as gender, age, number of diseases (co-morbidity), and care dependency as well as medical diagnosis. Care dependency is assessed with the care dependency scale (CDS) [24]. Furthermore, nutritional indicators such as weight, height, body mass index (BMI), and malnutrition risk are measured. We used two definitions for malnutrition risk. The first definition uses BMI and unintentional weight loss: malnutrition is indicated by a BMI <18.5 kg/m^2^ (in patients ≥65 years old ≤20 kg/m^2^) and/or unintended weight loss of >10%, respectively, within the last six months or >5% within the last month. The second definition of malnutrition is based on the risk as measured using the malnutrition universal screening tool (MUST). This tool is a widely recognized, valid, and reliable instrument for measuring malnutrition in hospitalized patients [25,26]. Obesity is defined as BMI ≥30 and underweight as BMI <18.5, according to the classification of the World Health Organization (WHO) [27]. We also determined if the patient had been screened for malnutrition risk upon admission to hospital, needed support while eating and drinking, or was referred to a dietitian during the hospital stay.

### 2.3. Data Collection

Participation in this study was voluntary for the hospitals. The hospitals were motivated to participate with all wards. Each hospital participating in the LPZ nominates an institutional coordinator who receives training on how to manage the measurement. Data are collected from patients by two healthcare professionals. The research team provides training sessions as well as written manuals to these persons, ensuring the collection of standardized data in all participating hospitals, and increasing the data reliability. All data were entered either directly into a specially designed and password-protected, web-based data-entry program or after their collection using printed paper questionnaires.

### 2.4. Subjects

All adult patients (18 yr+) that were admitted to the participating wards of the hospitals in 2017, 2018, or 2019 were asked to take part in the study (*n* = 16,599). Of these patients, 73% (*n* = 12,174) agreed to participate in the study. The final sample consisted of 8405 patients, for whom all data on BMI and unintentional weight loss were available.

### 2.5. Ethical Considerations

This study was approved by the ethics committee of the local university (20–192 ex 08/09). Patients or their legal representatives were informed of the study and had to provide their written informed consent. Hospital participation was voluntary. The study was performed in accordance with the ethical standards of the Declaration of Helsinki and its later amendments.

### 2.6. Statistical Analysis

To analyze data, we used the IBM SPSS Statistics for Windows software package, version 26 (IBM Corp., Armonk, N.Y., USA). We performed data cleaning and excluded patients with missing values (e.g., BMI and/or unintentional weight loss) from the analysis. Descriptive statistics were carried out, and dichotomous data are presented as absolute and relative frequencies. Metric data are shown as mean values with standard deviations (SD) or median values with interquartile ranges. To calculate the associations between the patient characteristics and dietitian referrals, we took a three-step approach. First, we conducted a univariate analysis using chi-squared tests and Mann–Whitney U tests, depending on the variable, to identify significant predictors for dietitian referral. Variables were chosen based on the literature and the researcher’s (DE) clinical experiences. We conducted tests to assess multicollinearity for all influencing variables based on a variance inflating factor (VIF), all of which were less than four. Second, we performed a univariate logistic regression and, third, we used a generalized estimating equation (GEE), with all variables that were significantly associated with dietitian referrals in the univariate regression analysis. The GEE is a useful method that can be applied to estimate binary outcomes (dietitian referral yes/no) when clustered data are used (different years of measurement and different institutions). All tests were two-sided; the significance level was set at 0.05.

## 3. Results

In this study, 45 different hospitals participated. Most of the 8405 participating patients were admitted to various kinds of internal wards (45.1%) or to surgical wards (33.5%). A further 9.6% were admitted to geriatric wards, 5.9%, to psychiatric wards, 1% were ICU patients, and 4.9% were admitted to other wards. The median age of the patients was 69, and 51.9% of the patients were female. According to the MUST, 23.3% of the patients were at risk of malnutrition, and 15.3% were at risk according to the definition that combined BMI and weight loss, whereas 22.3% were obese (BMI ≥30 kg/m^2^). Of all patients at risk of malnutrition, 16.8% were referred to a dietitian. Detailed patient characteristics are shown in Table 1.

The univariate binary logistic regression analysis results show that all variables included in the analysis, except for age and gender, are predictors for dietitian referrals. In the final multivariate GEE model, the following predictors remained significant: diabetes diagnosis (*OR* 1.80), cancer diagnosis (*OR* 1.76), digestive disease diagnosis (*OR* 2.03), presence of a pressure injury (*OR* 1.58), risk of malnutrition based on BMI and weight loss (*OR* 1.72), risk of malnutrition based on MUST (2.55), and the application of any malnutrition screening at admission to hospital (2.20). Table 2 shows the detailed results of the univariate and multivariate statistical analyses.

**Figure 1:**
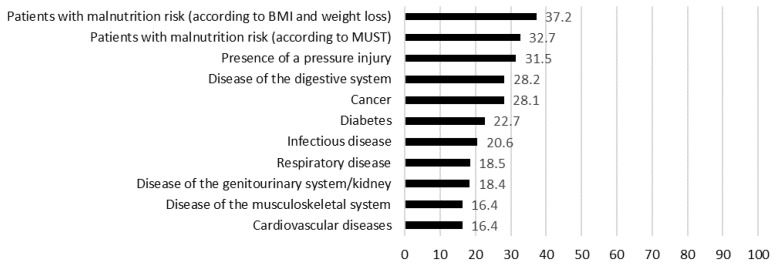
Frequency of dietitian referrals in different medical conditions; MUST = malnutrition universal screening tool; BMI = body mass index.

The highest rate of dietitian referrals was found in patients with a risk of malnutrition (37.2 and 32.7, depending on the underlying definition of malnutrition risk). Out of patients with pressure injuries, 31.5% were referred to a dietitian. The dietitian rates in different diseases that require the implementation of nutritional therapy delivered by dietitians are shown in Figure 1.

## 4. Discussion

This study included a large sample of hospitalized adult patients and revealed a low dietitian referral rate among these patients (i.e., only 16.8%). An existing risk of malnutrition was identified as the most important predictor for a dietitian referral. Patients who were at risk of malnutrition were referred more than 2.5 times more frequently to a dietitian than patients who were not at risk of malnutrition. Medical diagnoses such as diabetes, cancer, or digestive disorders were also identified as strong predictors for being referred to a dietitian. Other diseases such as kidney diseases and other indicators of nutritional status such as BMI, being underweight, or obesity were not associated with dietitian referrals. Age, gender, the degree of care dependency, or the number of medical diagnoses had no significant impact on the involvement of dietitians in patient care.

Even if malnutrition risk was a strong predictor for a dietitian referral, only about one-third of the patients at risk of malnutrition were referred to a dietitian. This is a rather low percentage considering that recognised guidelines (e.g., the European Society for Clinical Nutrition and Metabolism (ESPEN) or the American Society for Parenteral and Enteral Nutrition (ASPEN) guidelines) recommend that all patients at risk of malnutrition should be offered individualized nutritional counselling and interventions delivered by a qualified dietitian [8,28,29,30,31,32].

The existing literature clearly shows that dietitian involvement has considerable benefits for hospitalized patients, resulting in better health outcomes and improved quality of life [18,19,20,33,34]. For patients with obesity, for example, every Euro invested in dietary counseling by dietitians saves 14 to 63 Euros over five years [35]. Dietitians appear to be more effective in counselling compared to other healthcare specialists. However, there are still barriers to being referred to a qualified dietitian, and in some countries there are problems with reimbursement for dietitian services, especially in the outpatient setting [35,36]. This reveals and emphasizes the fact that improving dietitian involvement in the care of patients depends, amongst other things, also to a great extent on the respective country’s referral system and therefore its entire healthcare system.

It must be mentioned that the referral to a dietitian does not automatically result in better patient outcomes. Nutritional care and dietary counselling must be adapted to the respective patient’s individual needs and social situation, and multidisciplinary cooperation between all healthcare professionals within the clinical team is key for successful nutritional care. Furthermore, the patients’ adherence to their nutritional recommendations is an important factor for success [15,35].

Our study results show that patients who were screened for malnutrition risk at admission to hospital had a more than two-fold chance of receiving a dietitian visit compared to patients who were not screened. These study results confirm the importance of malnutrition screening in the hospital setting, particularly considering that the literature shows that a risk of malnutrition in hospitalized patients often goes undetected and, therefore, untreated if no screening tool is used [14,37]. Other studies have shown that more than half of the patients at risk of malnutrition are not identified when not applying a malnutrition screening routine [14,38,39]. Patients admitted to wards that do not use a malnutrition screening tool receive significantly fewer nutritional interventions than patients on wards using a screening tool [40]. Our results suggest that increasing the screening rate concurrently increases the number of patients with dietitian referrals and may further improve nutritional care in the respective hospitals.

Medical diagnoses that served as predictors of dietitian consultations in our study sample were diabetes mellitus, cancer, and digestive disorders. There is no question that these are nutrition-related diseases that require the involvement of dietitians. However, an examination of the dietitian referral rates revealed that only about one-quarter of patients with one of these diagnoses are referred to a dietitian. This means that three-quarters of the patients with one of these nutrition-related diseases never have an opportunity to receive counselling from a dietitian during their hospital stay. These findings demonstrate that the current dietitian referral systems are inadequate in Austria. In cancer patients, for example, this system can have serious negative consequences, such as negative health outcomes and diminished quality of life, since it is known that patients who receive counselling from qualified dietitians show positive improvements in their nutritional intake, nutritional status, and quality of life [34].

This study provides valuable insights into current dietitian referral practices in hospitals. However, this study also has limitations. The study sample was a convenient sample of Austrian hospitals, and hospital participation in the study was voluntary. Therefore, participating hospitals may have paid more attention to the quality of patient care than non-participating hospitals. Of patients admitted to the hospitals on the day of measurement, 73% voluntarily chose to take part in the study and fully completed the questionnaire. Patients who chose not to participate were often in a poor overall state of health, and this could have biased the dietitian referral rates. Finally, we chose predictors for dietitian referrals on the basis of our literature review and personal experience. Other important predictors for dietitian referrals might exist that were not included in our analysis.

## 5. Conclusions

The number of patients with nutrition-related diseases and health conditions that receive a dietitian consultation during their hospital stay is rather low and urgently needs to be increased. This may be achieved by implementing mandatory malnutrition screenings in hospitals. Adequately staffing hospitals with dietitians would also allow an increased number of dietitian referrals to be made. The development of dietitian referral criteria may facilitate the involvement of dietitians in the healthcare process, since there is often uncertainty among staff as to which person should be referred to a dietitian and when this should happen. Furthermore, to increase the dietitian referral rates of hospitalized patients, dietitians must be constantly involved in the patient healthcare process, in order to raise awareness among physicians and other healthcare professionals regarding the impact of this involvement in the holistic and multidisciplinary treatment of hospital patients.

## Figures and Tables

**Table 1:** Patient characteristics for all patients, as well as for patients with and without a dietitian referral.

	Total *N* = 8405	Dietitian Referral YES *n* = 1416	Dietitian Referral NO *n* = 6989	*p* -Value
Demographic variables				
Age, median (IQR)	69 (55–79)	68 (56–78)	69 (55–79)	0.03
Gender, female, *n* (%)	4359 (51.9)	720 (50.8)	3639 (52.1)	0.40
CDS sum score, median (IQR)	74 (64–75)	71 (57–75)	74 (65–75)	0.00
Medical diagnoses				
Number of medical diagnoses, median (range)	2 (1–4)	3 (2–4)	2 (1–4)	0.00
Cardiovascular disease, *n* (%)	3941 (46.9)	648 (45.8)	3293(47.1)	0.35
Disease of the musculoskeletal system/connective tissue, *n* (%)	2244 (26.7)	368 (26.0)	1876 (26.8)	0.51
Urogenital tract disease, incl. kidney, *n* (%)	1863 (22.2)	343 (24.2)	1520 (21.7)	0.04
Digestive disease diagnosis, *n* (%)	1809 (21.5)	511 (36.1)	1298 (18.6)	0.00
Respiratory diagnosis, *n* (%)	1776 (21.1)	329 (23.2)	1447 (20.7)	0.03
Cancer diagnosis, *n* (%)	1512 (18.0)	425 (30.0)	1087 (15.6)	0.00
Diabetes diagnosis, *n* (%)	1475 (17.5)	335 (23.7)	1140 (16.3)	0.00
Infectious diseases, *n* (%)	562 (6.7)	116 (8.2)	446 (6.4)	0.01
Presence of a pressure injury, *n* (%)	222 (2.7)	70 (5.0)	152 (23–30)	0.00
Nutrition-related variables				
BMI, median (IQR)	25.6 (23–29)	24.1 (21–28)	25.9 (23–30)	0.00
Obesity, *n* (%)	1871 (22.3)	261 (18.4)	1610 (23.0)	0.00
Underweight, *n* (%)	360 (4.3)	155 (10.2)	216 (3.1)	0.00
Risk of malnutrition (BMI and weight loss), *n* (%)	1286 (15.3)	478 (33.8)	808 (11.6)	0.00
Risk of malnutrition (MUST), *n* (%)	1858 (23.3)	608 (47.5)	1250 (18.7)	0.00
Screened for malnutrition risk, *n* (%)	4183 (49.8)	929 (65.6)	3254 (46.6)	0.00
Need for support during eating and drinking, *n* (%)	1431 (17.0)	361 (25.5)	1070 (15.3)	0.00

IQR = Interquartile range; *p*-values are shown for differences between patients with and without a dietitian referral, using chi-squared tests or Mann-Whitney *U* Tests, depending on the distribution of the data; care dependency as assessed with the Care Dependency Scale (CDS); possible scores range between 15 and 75, whereby lower scores indicate a higher care dependency; MUST Malnutrition Universal Screening Tool; BMI Body Mass Index.

**Table 2:** Predictors for dietitian referral, univariate binary logistic regression (BLR), and multivariate generalized estimating equation model (GEE).

	Univariate Analysis (BLR)			Multivariate Analysis (GEE)		
	Odds Ratio	95% CI	*p*-Value	Odds Ratio	95% CI	*p*-Value
Demographic variables						
Age	1.00	1.00–1.00	0.12			
Gender, female	1.00	0.85–1.07	0.40			
Care Dependency (sum score)	1.00	0.98–0.99	0.00	1.00	0.99–1.00	0.27
Medical diagnoses						
Number of medical diagnoses	1.18	1.15–1.22	0.00	1.04	0.99–1.10	0.10
Diabetes diagnosis	1.59	1.39–1.83	0.00	1.80	1.50–2.16	0.00
Cancer diagnosis	2.33	2.04–2.65	0.00	1.76	1.51–2.05	0.00
Digestive disease diagnosis	2.48	2.19–2.80	0.00	2.03	1.75–2.37	0.00
Urogenital tract disease incl. kidney	1.15	1.00–1.32	0.04	0.95	0.80–1.12	0.52
Respiratory diagnosis	1.16	1.01–1.03	0.03	1.11	0.94–1.31	0.24
Infectious diseases	1.31	1.06–1.62	0.01	0.90	0.70–1.15	0.38
Presence of a pressure injury	2.35	1.76–3.13	0.00	1.58	1.10–2.27	0.01
Nutrition-related variables						
BMI	0.95	0.94–0.96	0.00	0.99	0.97–1.02	0.49
Obesity	0.76	0.65–0.87	0.00	0.94	0.73–1.22	0.66
Underweight	3.55	2.85–4.42	0.00	1.05	0.77–1.43	0.77
Risk of malnutrition (BMI and weight loss)	3.90	3.42–4.45	0.00	1.72	1.38–2.15	0.00
Risk of malnutrition (MUST)	3.94	3.48–4.47	0.00	2.55	2.15–3.02	0.00
Screened for malnutrition risk	2.19	1.94–2.47	0.00	2.18	1.91–2.50	0.00
Need for support during eating and drinking, *n* (%)	1.89	1.65–2.17	0.00	0.79	0.61–1.01	0.06

BMI = body mass index, MUST = malnutrition universal screening tool.
